# Knockout of *Katnal2* Leads to Autism-like Behaviors and Developmental Delay in Zebrafish

**DOI:** 10.3390/ijms23158389

**Published:** 2022-07-29

**Authors:** Jing Zheng, Fei Long, Xu Cao, Bo Xiong, Yu Li

**Affiliations:** 1 Department of Forensic Medicine, Tongji Medical College, Huazhong University of Science and Technology, Wuhan 430030, China; d201981451@hust.edu.cn (J.Z.); d201881334@hust.edu.cn (F.L.); caoxu@hust.edu.cn (X.C.); 2 School of Basic Medicine, Tongji Medical College, Huazhong University of Science and Technology, Wuhan 430030, China; 3 Department of Neurosurgery, Tongji Hospital, Tongji Medical College, Huazhong University of Science and Technology, Wuhan 430030, China

**Keywords:** *katnal2*, zebrafish, neurodevelopmental disorder, autism spectrum disorder, brain development, behavior

## Abstract

*KATNAL2* mutations have been associated with autism spectrum disorder (ASD) and other related neurodevelopmental disorders (NDDs) such as intellectual disability (ID) in several cohorts. *KATNAL2* has been implicated in brain development, as it is required for ciliogenesis in *Xenopus* and is required for dendritic arborization in mice. However, a causative relationship between the disruption of Katnal2 function and behavioral defects has not been established. Here, we generated a *katnal2* null allele in zebrafish using CRISPR/Cas9-mediated genome editing and carried out morphological and behavioral characterizations. We observed that *katnal2^-/-^* embryos displayed delayed embryonic development especially during the convergence and extension (CE) movement. The hatched larvae showed reduced brain size and body length. In the behavioral tests, the *katnal2^-/-^* zebrafish exhibited reduced locomotor activity both in larvae and adults; increased nocturnal waking activity in larvae; and enhanced anxiety-like behavior, impaired social interaction, and reduced social cohesion in adults. These findings indicate an important role for *katnal2* in development and behavior, providing an in vivo model to study the mechanisms underlying the ASD related to *KATNAL2* mutations.

## 1. Introduction

Autism spectrum disorder (ASD) represents a group of genetically and clinically heterogeneous neurodevelopmental disorders. The hallmarks of ASD mainly include social communication deficits, restricted interests, and repetitive behavior [1,2]. The global prevalence of ASD has increased steadily in recent years, and it is the leading psychiatric disorder in children under 5 years of age [3]. Currently, the pathogenesis of ASD remains poorly understood. The causes of ASD are complex and include both genetic variants and environmental factors, in which the genetic factors account for up to 80% of disease occurrence [4]. Sequencing projects of large ASD cohorts have identified many candidate genes associated with ASD; however, further studies are required to pinpoint their functions in brain development and behavior [5].

In humans, Katanin Catalytic Subunit A1 Like 2 (*KATNAL2*, NCBI gene ID: 83473) is located on chromosome 18q21.1. There have been a number of studies showing that *KATNAL2* is an ASD risk gene. Initially, two de novo mutations in the *KATNAL2* gene were separately reported in autistic probands in the Simons simplex collection [6,7]. Subsequently, *KATNAL2* was identified as a statistically significant candidate gene for ASD (FDR ≤ 0.01) as more de novo variants were reported [8,9]. The association between *KATNAL2* and nervous system development is then repeatedly validated by sequencing studies in various populations [6,7,8,9,10,11,12,13,14,15]. In total, 49 mutations in the *KATNAL2* gene (27 missense and 22 loss of function mutations) were identified in patients with ASD and neurodevelopmental disorders (NDDs).

KATNAL2 is proposed to be a microtubule (MT)-severing ATPase based on structural prediction. It has been reported that KATNAL2 plays important roles in cellular processes involving cytokinesis, MT dynamics, cell-cycle progression and ciliogenesis [16,17,18]. It is known that microtubule severance and transport play a critical role in neuron formation [19]. Nevertheless, there has been a limited number of studies related to KATNAL2 function in the nervous system. In neonatal mouse, knockout of *Katnal2* leads to decreased dendritic arborization [20]. In *Xenopus* embryos, morpholino-mediated knockdown or genetic knockout of *katnal2* causes abnormalities during embryonic development and organogenesis, including reduced brain and eye size [17]. In addition, two other Katanin proteins, KATNAL1 and KATNB1, were also found to affect the development of the nervous system [21,22,23]. These observations suggest that KATNAL2 is essential for early brain development. However, the role of KATNAL2 regarding animal behaviors has not been examined in previous models.

Zebrafish *katnal2* shares 56% identity with human *KATNAL2*. The zebrafish Katnal2 protein contains the same major functional domain as the human homolog. Therefore, it is feasible to use zebrafish as a model organism to study the function of *katnal2* gene. To examine the impact of *katnal2* loss on behaviors, especially these closely related to ASD, we generated a *katnal2* loss-of-function mutation in zebrafish using the CRISPR/Cas9 gene-editing technology and then performed a series of morphological analyses and behavioral tests. We found that the *katnal2^-/-^* zebrafish exhibited developmental delay, microcephaly, a disturbed sleep pattern, elevated anxiety level and impaired social behaviors, which are reminiscent of key ASD characteristics. These data establish a causative relationship between *katnal2* loss and behavioral defects, providing an in vivo model for further basic and translational studies.

## 2. Results

### 2.1. Generation of Katnal2^-/-^ Zebrafish

**Figure 1:**
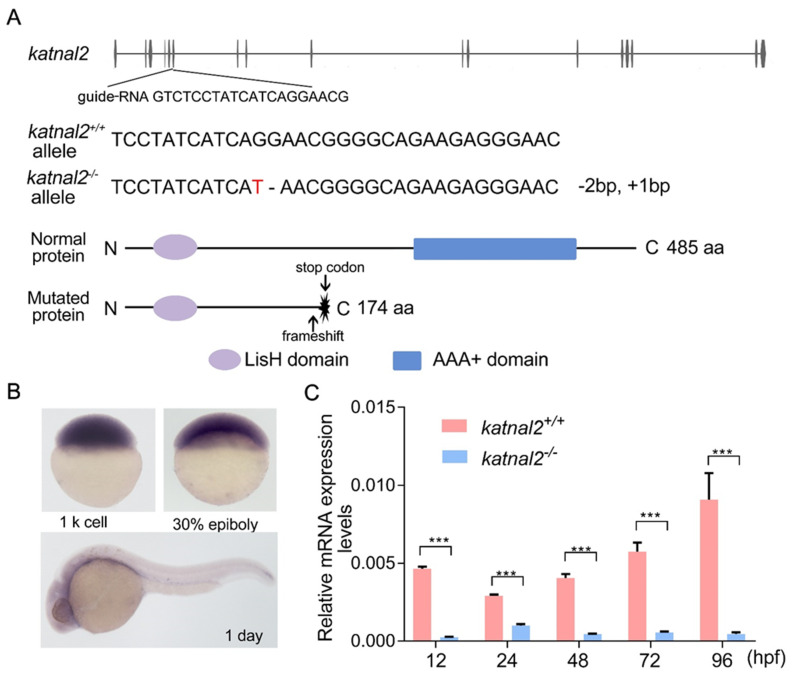
Generation of *katnal2*^-/-^ allele by CRISPR/Cas9 gene-editing technology. (**A**) Structure of zebrafish *katnal2* gene. The protein includes an N-terminal LisH (Lis-homology) domain and a C-terminal AAA catalytic domain. A guide-RNA was designed to target a 23 bp region in exon 6. The *katnal2*^-/-^ allele contains a 2-base deletion and a 1-base insertion. The red color indicates the inserted nucleotides and the “-” indicates the deleted nucleotides. The mutation causes frameshift and early truncation of the protein prior to the AAA+ domain. (**B**) The expression pattern of *katnal2* in the early embryonic development determined by in situ hybridization. (**C**) The expression level of *katnal2* mRNA in *katnal2*^+/+^ (pink) and *katnal2*^-/-^ (blue) alleles. Data are shown as mean ± S.E.M.; *** indicates *p* < 0.001.

To examine the role of *katnal2* in regulating animal behavior, we generated a zebrafish knockout model using the CRISPR/Cas9 system (Figure 1A). We first determined the temporal and spatial expression pattern of *katnal2* by in situ hybridization. *katnal2* is maternally expressed at the early embryonic stage and enriched in the developing brain at 24 h post-fertilization (hpf), indicating that it may function in neurodevelopment (Figure 1B). We designed and tested three guide RNAs and eventually selected the one targeting a 23-base sequence in exon 6. Using an F3 screening strategy, we isolated a *katnal2* mutant allele carrying a deletion of 2 bp and an insertion of 1 bp, resulting in frameshift and truncation of the protein at amino acid 174. The mutation abolished the important AAA+ domain of the Katnal2 protein (Figure 1A). Moreover, the mRNA levels of *katnal2* were significantly decreased in mutant zebrafish larvae at various stages, suggesting that the mutation induced nonsense-mediated decay of the *katnal2* mRNA (Figure 1C). Thus, these results indicate that we successfully generated a genetic knockout allele of *katnal2* (*katnal2*^-/-^).

### 2.2. Katnal2^-/-^ Zebrafish Displayed Developmental Delay during Embryogenesis

**Figure 2:**
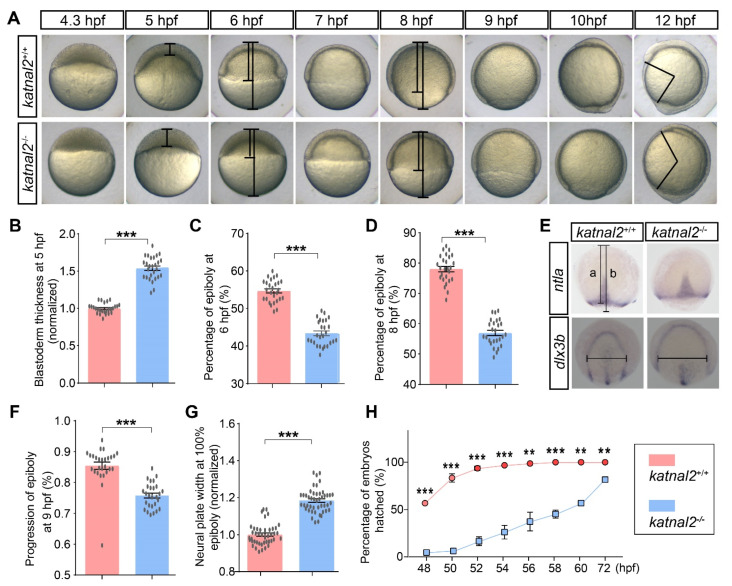
*Katnal2^-/-^* embryos exhibited developmentally delayed phenotypes. (**A**) Images of the embryos in time series from the initiation until the finish of epiboly. Images are lateral views with the animal pole on the top. (**B**) Normalized blastoderm thickness of the *katnal2^+/+^* and *katnal2^-/-^* embryos at 5 hpf (+/+, N = 26; -/-, N = 27). (**C**,**D**) The epiboly progression at 6 hpf and 8 hpf (N = 27 for each genotype) measured as indicated in panel (**A**). (**E**) Expression patterns of *ntla* in 9 hpf embryos and *dlx3b* in 100% epiboly embryos were analyzed by in situ hybridization. The line “**a**” represents the distance from the animal pole to the boundary of epiboly movement. The line “**b**” represents the distance between the animal and the plant pole. The horizontal line in *dlx3b* staining results represents the width of the neural plate. (**F**) *katnal2*^-/-^ embryos exhibited epiboly movement delay at 9 hpf as determined by *ntla* staining (N = 27 for each genotype). (**G**) Normalized neural plate width determined by *dlx3b* staining at 100% epiboly stage (+/+, N = 43; -/-, N = 46). (**H**) Percentage of embryos hatched was recorded every 2 h from 48 hpf to 60 hpf. Data are shown as mean ± S.E.M.; ** indicates *p* < 0.01, and *** indicates *p* < 0.001.

**Figure 3:**
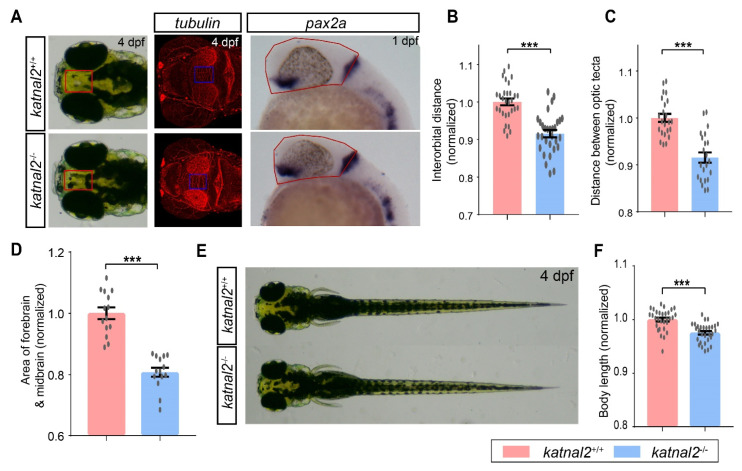
*Katnal2*^-/-^ larvae displayed morphological changes. (**A**) Illustrations of the measurements of the distance between the eyes, the distance between the optic tecta, and the area of forebrain and midbrain. (**B**) Comparisons of the normalized interorbital distance of 4 dpf *katnal2*^+/+^ and *katnal2*^-/-^ larvae (N = 30 for each genotype). (**C**) Normalized width between optic tecta of 4 dpf larvae (+/+, N = 23; -/-, N = 22). (**D**) Normalized area of the forebrain and midbrain region for 1 dpf embryos as determined by *pax2a* staining (N = 14 for each genotype). (**E**,**F**) Comparison of the body length of 4 dpf *katnal2*^+/+^ and *katnal2*^-/-^ larvae (N = 30 for each genotype). Data are shown as mean ± S.E.M.; *** indicates *p* < 0.001.

We first examined the role of *katnal2* during embryonic development by morphological assessment of *katnal2*^-/-^ and time-matched *katnal2*^+/+^ embryos. The *katnal2*^-/-^ embryos exhibited an obvious developmental delay phenotype. At 4.3 hpf, when epiboly was initiated in wild-type embryos with the yolk doming into the blastoderm, there was still a flat border between the blastoderm and the yolk in *katnal2*^-/-^ embryos (Figure 2A). At 5 hpf, the blastoderm became thinner in *katnal2*^+/+^ embryos, whereas it was much thicker in *katnal2*^-/-^ embryos (Figure 2A,B). From 6 to 10 hpf, the epiboly progression in *katnal2*^-/-^ embryos was also delayed, judged by the slowed spreading of the blastoderm toward the vegetal pole (Figure 2A,C,D). As a result, when *katnal2^+/+^* embryos completed gastrulation at 10 hpf, *katnal2^-/-^* embryos only reached at about 90% epiboly (Figure 2A). At 12 hpf, all *katnal2^-/-^* embryos completed epiboly and presented a shortened anteroposterior axis compared to time-matched *katnal2^+/+^* embryos (Figure 2A). Loss of *katnal2* not only affected epiboly but also affected the convergence and extension (CE) movements (Figure 2E–G). Upon completion of epiboly, the width of the neural plate of *katnal2^-/-^* embryos was significantly wider than *katnal2^+/+^* embryos, as determined by *dlx3b* mRNA expression (Figure 2E,G). Moreover, the *katnal2^-/-^* embryos hatched later than the *katnal2^+/+^* embryos (Figure 2H). Subsequently, we measured the body length, distance between the eyes, distance between the optic tecta, and area of forebrain and midbrain at 4 dpf to assess the brain development upon loss of *katnal2*. Compared to *katnal2^+/+^* larvae, the body length, width between the eyes, distance between the optic tecta, and area of forebrain and midbrain of *katnal2^-/-^* zebrafish were significantly decreased (Figure 3A–F). Taken together, these data indicate that *katnal2* is essential for proper embryonic and larval development in zebrafish. 

### 2.3. Katnal2^-/-^ Larvae Displayed Abnormal Sleep States and Impaired Locomotion Activity

**Figure 4:**
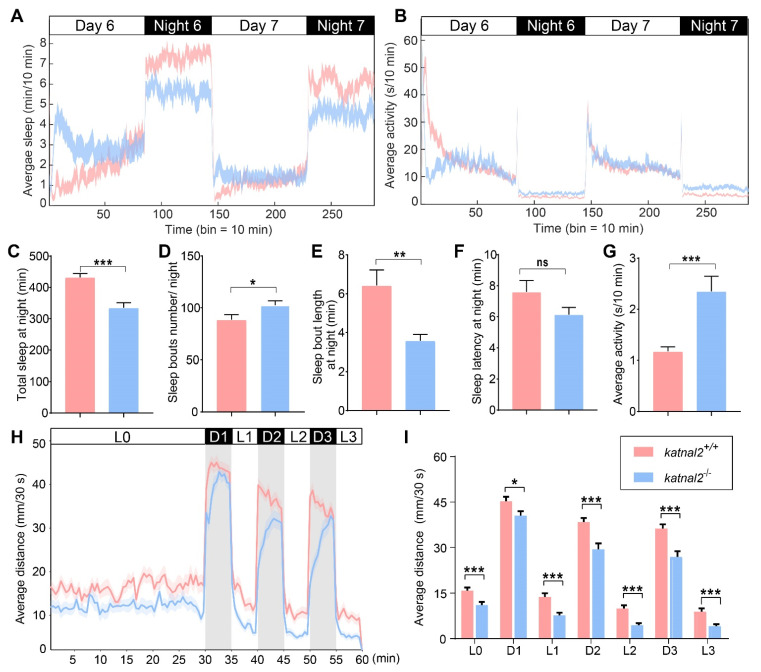
*Katnal2^-/-^* larvae displayed abnormal sleep/awake patterns and impaired locomotion activity. (**A**) Time sequence diagram of average sleep over time (N = 48 for each genotype). White and black bars above behavioral traces indicate the day (14 h) and night (10 h), respectively. (**B**) Time sequence diagram of average total activity over time. (**C**) Total sleep length on the sixth night. (**D**) Sleep bouts number on the sixth night. (**E**) Sleep bouts duration on the sixth night. (**F**) Sleep latency after light-off on the sixth night. (**G**) Average total waking activity on the sixth night. (**H**) The average distance moved within each 30 s bin under either light or dark conditions is plotted (N = 48 for each genotype). White and black bars above the behavioral traces indicate light (30 min or 5 min) and dark (5 min) conditions, respectively. (**I**) The average distance moved in 30 s during 30 min of light and three 5-min light/dark intervals. Data are shown as mean ± S.E.M.; * indicates *p* < 0.05, ** indicates *p* < 0.01, *** indicates *p* < 0.001 and ns indicates no significance.

Since human *KATNAL2* mutations have been associated with ASD, we sought to determine whether the loss of function of *katnal2* leads to alterations in behaviors. We first examined larval circadian rhythms by measuring sleep length, sleep bouts, and average waking activity under day–night cycles for 2 days between 5 and 7 dpf. Remarkably, *katnal2^-/-^* larvae showed a decreased average sleep length, increased sleep bouts, and decreased sleep length per bout at night (Figure 4A,C–E). However, the sleep latency during the day–night switch was not significantly altered in *katnal2^-/-^* larvae (Figure 4F). The average waking activity of *katnal2^-/-^* larvae was significantly higher than that of *katnal2*^+/+^ larvae at night, whereas it was comparable to that of *katnal2*^+/+^ larvae during the daytime (Figure 4B,G). These data show that the loss of *katnal2* causes sleep disturbance.

We also examined the responses evoked by short-time light changes (light/dark switch, 100 lx for brightness, and 0 lx for dark). After a 30 min habituation period, each larva was recorded for 30 min over three light/dark cycles (each consisting of 5 min in light and 5 min in dark setting per cycle). Compared with *katnal2*^+/+^ larvae, *katnal2^-/-^* larvae moved significantly shorter total distances in all periods during the test (Figure 4H,I). The transition from light to dark caused a sudden increase in movement velocity, while the transition from dark to light caused a sudden decrease. *katnal2^-/-^* larvae showed a delayed response pattern to changes in illumination (Figure 4H). These data suggest that loss of *katnal2* impairs zebrafish larval locomotion activity and attenuates the response to stimuli. 

### 2.4. Adult Katnal2^-/-^ Zebrafish Displayed Impaired Locomotion Activity and Elevated Anxiety Level

**Figure 5:**
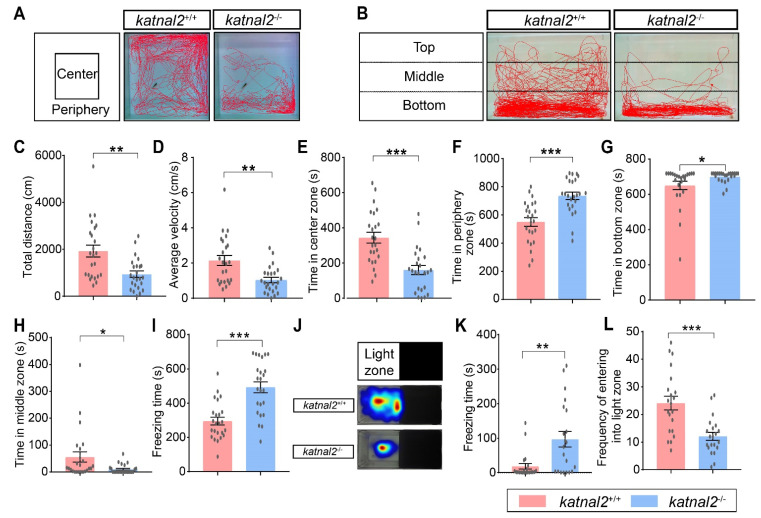
Adult *katnal2^-/-^* zebrafish displayed reduced locomotion activity and anxiety-like behaviors. (**A**) Schematic diagram of the open-field test and representative trajectory diagrams (N = 24 for each genotype). (**B**) Schematic diagram of the novel tank test and representative trajectory diagrams (N = 24 for each genotype). (**C**) The total distance moved in the open field test. (**D**) The average velocity in the open field test. (**E**) Duration time in the center zone. (**F**) Duration time in the peripheral zone. (**G**) Duration time in the bottom zone in the novel tank test. (**H**) Duration time in the middle zone in the novel tank test. (**I**) The freezing time in the novel tank test. (**J**) Schematic diagram of the light-and-dark test and heat map of sojourn time (N = 21 for each genotype). (**K**) The freezing time in the light zone. (**L**) The frequency of the tester zebrafish entering the light zone. Data are shown as mean ± S.E.M.; * indicates *p* < 0.05, ** indicates *p* < 0.01, and *** indicates *p* < 0.001.

The *katnal2^-/-^* allele is viable and fertile, allowing behavioral tests in the adult stage. We performed an open field test to examine the locomotion activity and anxiety level of adult zebrafish (Figure 5A). A significant reduction in the total distance and swimming velocity was observed in *katnal2^-/-^* zebrafish compared to *katnal2*^+/+^ (Figure 5C,D). We further calculated the time spent in the central and peripheral zone for both genotypes. Compared to the controls, the *katnal2^-/-^* spent less time in the central zone and more time in the peripheral zone, indicating an elevated anxiety level (Figure 5E,F).

To further confirm that loss of *katnal2* causes higher anxiety, we also performed the novel tank test and the light-and-dark box test. When dropped to a novel tank, we found that *katnal2^-/-^* zebrafish spent more time in the bottom zone and less time in the middle zone than *katnal2*^+/+^ zebrafish, again suggesting an elevated anxiety level (Figure 5B,G,H). Similar to the result of the open field test, *katnal2^-/-^* zebrafish displayed reduced total distance traveled and swimming velocity in the novel tank. In addition, *katnal2^-/-^* zebrafish displayed more freezing time than *katnal2*^+/+^ zebrafish (Figure 5I). In the light-and-dark box test, *katnal2^-/-^* zebrafish displayed a significantly lower frequency of swimming from the dark zone into the light zone and more freezing time in the light zone (Figure 5J–L). Taken together, these data suggest that *katnal2* loss of function mutation leads to lower locomotion activity and a higher anxiety level, which correlates with the observations in the larvae. 

### 2.5. Katnal2^-/-^ Zebrafish Exhibited ASD-like Behaviors 

**Figure 6:**
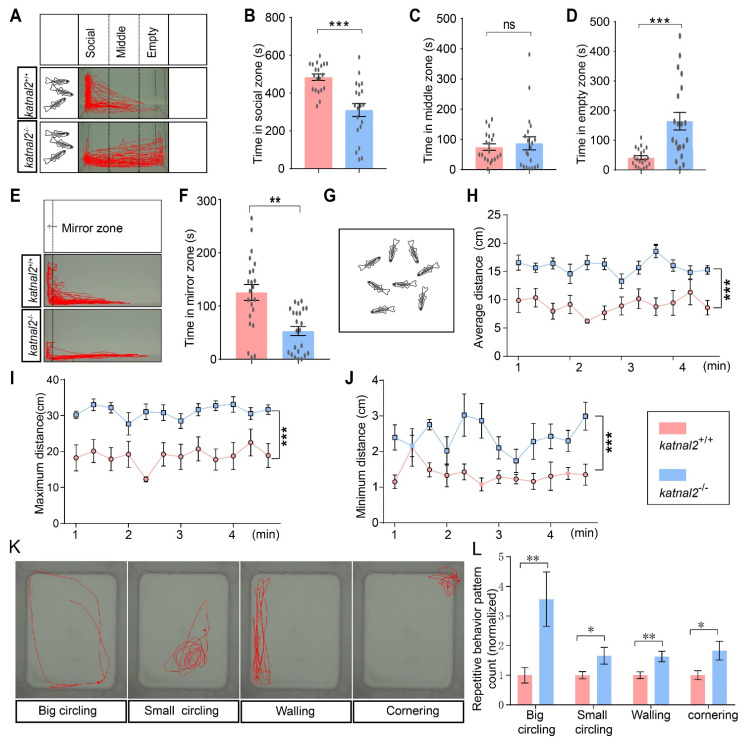
*Katnal2^-/-^* zebrafish showed impaired social interaction and reduced social cohesion. (**A**) Schematic diagram of the three-tank social interaction test and representative trajectory diagrams (N = 20 for each genotype). (**B**) Duration time in the social zone. (**C**) Duration time in the middle zone. (**D**) Duration time in the empty zone. (**E**) Schematic diagram of the mirror test and representative trajectory diagrams (+/+, N = 22; -/-, N = 23). (**F**) Duration time in the mirror zone. (**G**) Schematic diagram of the shoaling test (N = 9 for each genotype). (**H**) The average inter-fish distance was recorded and calculated every 20 s. (**I**) The maximum inter-fish distance was recorded and calculated every 20 s. (**J**) The minimum inter-fish distance was recorded and calculated every 20 s. (**K**) Representative trace of big circling, small circling, and walling. (**L**) Comparison of the frequency of different repetitive behaviors between *katnal2* mutants and controls (N = 45 for each phenotype). Data are shown as mean ± S.E.M.; * indicates *p* < 0.05, ** indicates *p* < 0.01, and *** indicates *p* < 0.001 and ns indicates no significance.

Deficit in social communications is one of the key features of ASD; therefore, we examined the social activities of the *katnal2^-/-^* allele. In the three tank tests, *katnal2*^+/+^ zebrafish spent most of the time in the social zone and were only occasionally present in the middle or empty zone. In contrast, *katnal2^-/-^* zebrafish spent significantly less time in the social zone and comparatively more time in the middle and empty zone (Figure 6A–D). In the mirror test, the *katnal2^-/-^* zebrafish spent less time in the mirror zone where they could approach their own reflections (Figure 6E,F). Taken together, these data suggest that *katnal2^-/-^* zebrafish show reduced interest in social interaction.

Zebrafish are natural schooling fish. To test the group behavior of the *katnal2*^-/-^ allele, we performed the shoaling test to assess the social cohesion among homogeneous groups of zebrafish. In this assay, adult *katnal2*^+/+^ or *katnal2*^-/-^ zebrafish were placed in the testing tank in groups of eight fish (Figure 6G). The average inter-fish distance, maximum inter-fish distance and minimum inter-fish distance was measured every 20 s for all pair combinations. *katnal2*^+/+^ zebrafish typically swam in the same direction and maintained a short inter-fish distance at all timepoints, whereas the *katnal2*^-/-^ zebrafish exhibited increased average inter-fish distance as a significant number of zebrafish swimming away from the group, showing a decreased social cohesion behavior (Figure 6H–J). Repetitive and stereotyped behavior is another core symptom of ASD. Compared to the controls, *katnal2^-/-^* zebrafish exhibited a significantly higher frequency of stereotypical behaviors, including repetitive big circling, small circling, walling and cornering (Figure 6K–L). Taken together, these data showed that knockout of katnal2 in zebrafish leads to phenotypes reminiscent of the core features of ASD.

## 3. Discussion

Although human *KATNAL2* has been identified as a candidate gene for ASD, so far, there have been no reported behavioral studies in *Katnal2* knockout animals. In this study, we generated a *katnal2* loss-of-function allele in zebrafish using the CRISPR/Cas9 gene-editing technology, revealed the morphological abnormity of *katnal2^-/-^* zebrafish at the early developmental stage, and characterized the behavioral changes of mutants both in larval stage and in adulthood. 

The *katnal2^-/-^* embryos exhibited delayed epiboly initiation and progression, resulting in reduced elongation of the anteroposterior axis. *Katnal2* is a paralogue of the microtubule severing enzymes *Katna1* and *Katnal1*; therefore, it is proposed to possess the microtubule-severing capacity [16,24]. KATNAL2 is associated with a variety of MT-dependent cellular processes [16]. *Katnal2* knockdown can result in defective ciliogenesis, inefficient cell division, increased cell and nuclear volume, formation of abnormal multipolar mitotic spindles, mitotic defects, increased MT acetylation, and cell-cycle alterations [16,17]. Furthermore, a previous study has shown that microtubule arrays formed in yolk syncytial layer are critical for driving vegetal migration of the yolk syncytial nuclei [25]. Hence, *katnal2* loss of function in mutant embryos may affect the structure and dynamics of microtubules, which are critical for cell migrations. We propose that the developmental delay of *katnal2^-/-^* embryos is due to the defects in the microtubule network. 

The decreased distance between the eyes, decreased distance between the optic tecta, and reduced area of forebrain and midbrain indicate that *katnal2* is essential for proper brain development, which is consistent with observations in the *katnal2* knockdown *Xenopus* model and *katnal2* knockout rodent model [17,20]. The role of *katnal2* in neurodevelopment and development control could relate to the role of *katnal2* in multiple MT-dependent cellular processes such as cell division and cell migration. It will be of great importance to further study the cellular mechanisms underlying the critical roles of *katnal2* in brain development. 

In recent years, the zebrafish has emerged as a valuable animal model in ASD research [26,27]. Many behavioral tests have been developed in zebrafish models, including the evaluation of social interaction, social cohesion, inhibitory avoidance, repetitive stereotyped behavior, environmental adaptation, the fear and anxiety response, novelty-seeking, and aggression [28,29]. We therefore performed behavioral tests and observed behavioral abnormalities in the *katnal2^-/-^* allele. The *katnal2^-/-^* larvae displayed impaired locomotor activity, which may be directly related to the developmental delay or neurodevelopment abnormalities. Interestingly, *katnal2^-/-^* larvae showed more fragmented sleep time and a marked increase in waking activity at night. The selectively enhanced nocturnal waking activity may suggest that *katnal2* deficiency caused an imbalance in excitatory and inhibitory signaling in mutant fish, which has been reported in a previous study [30]. In the open field test, *katnal2^-/-^* zebrafish displayed decreased distance moved, velocity, and time to explore the center zone. In the novel tank test, *katnal2^-/-^* zebrafish showed a reduction in distance moved and time spent in the middle zone as well as an increase in time spent in the bottom zone. In addition, the freezing time was also increased. In the three-tank social interaction test, the open field test, the shoaling test, and the mirror test, the *katnal2^-/-^* zebrafish exhibited core ASD-like behaviors, including impaired social interaction and cohesion as well as repetitive or stereotyped behaviors. Unfortunately, detailed phenotypical data were not reported for patients carrying *KATNAL2* mutations in large-scale sequencing projects. We cannot make a direct comparison between the patient symptoms and the zebrafish mutant phenotypes. However, the defects we observed, such as developmental delay, anxiety, locomotion defects and sleep disturbance, are typical co-morbidities besides the core features of ASD. Our findings could provide clues for clinicians to carefully and thoroughly examine the phenotypes of patients carrying *KATNAL2* mutations. Additionally, high-throughput drug-screening methods have been established for zebrafish larvae [31,32]. Since the *katnal2* mutants exhibit abnormal morphology and behaviors as early as the larval stage, it is feasible to perform large-scale screening of small molecules to identify therapeutic candidates. Therefore, the knockout model we generated would help facilitate further studies related to the molecular mechanism of Katnal2 and drug screening.

## 4. Materials and Methods 

### 4.1. Zebrafish Culture

Wild-type AB strain zebrafish were purchased from the China Zebrafish Resource Center. Adult fish were maintained in a water cycling system at 28.5 °C with a 14 h-light/10 h-dark circadian cycle. Embryos were raised in E3 medium in a 28.5 °C incubator. All zebrafish protocols used in this study were approved by the Animal Care and Use Committee of the animal core facility at Huazhong University of Science and Technology.

### 4.2. Generation of Katnal2 Mutant Zebrafish

The *katnal2^-/-^* zebrafish were generated using CRISPR/Cas9, as previously described [33,34]. A mixture of 600 pg of Cas9 mRNA and 80 pg of guide-RNA was microinjected into one cell stage of fertilized zebrafish eggs. Efficiencies of the guide-RNAs were validated by PCR and Sanger sequencing, and a guide-RNA targeting exon 6 (sequence, 5′-GTCTCCTATCATCAGGAACG-3′) was selected. The homozygous *katnal2^-/-^* was isolated in a standard F3 screen. The F3 *katnal2^+/+^* littermates were maintained and used as controls in all experiments. 

### 4.3. RNA Extraction and RT-PCR

Total RNA was extracted from whole embryos (50 embryos per sample) or larvae (50 larvae per sample) using Trizol reagent (Ambion, Austin, TX, USA) according to the manufacturer’s instructions. Reverse transcription was performed with a HiScript^®^III RT SuperMix (Vazyme, Nanjing, China). Oligo dT primer and random primer were added in 20 μL mixture to synthesize cDNA. Real-time PCR was performed using a lightCycler 96 apparatus (Roche, Basel, Switzerland) and ChamQ SYBR qPCR Master Mix (Vazyme, Nanjing, China), according to the instructions of the manufacturer. The delta-delta CT method was used to calculate the expression levels. RT-PCR was performed in triplicate. Primers used in real-time PCR are listed below.

*actb1*-F, 5′-GATGAGGAAATCGCTGCCCT-3′; 

*actb1*-R, 5′-ATGCCAACCATCACTCCCTG-3′; 

*katnal2*-F, 5′-TGGAGGGGAGACTCTGAGAA-3′;

*katnal2*-R, 5′-CACTGATTCCAGCTCATCCA-3′.

### 4.4. Whole Mount mRNA In Situ Hybridization

In situ hybridization was performed as previously described using the following digoxigenin (DIG RNA label kit, Roche, Basel, Switzerland)-labeled antisense probes: *katnal2, ntla, dlx3b, pax2a*. The embryos were fixed overnight in 4% paraformaldehyde (PFA) solution, dehydrated by increasing the gradient of methanol, and kept at −20 °C in a freezer. The whole-mount in situ hybridization was performed following a standard protocol [35]. After gradient rehydration in decreasing methanol series (75%, 50%, and 25%) in PBS and washing with PBS containing 0.1% Tween 20 (PBST) four times, the embryos were digested with proteinase K (10 μg/mL) and then hybridized with digoxigenin-labeled probes in hybridization mix (HM: 50% formamide, 5 × SSC, 50 μg/mL heparin, 5 mg/mL tRNA, 0.1% Tween 20) overnight after pre-hybridizing for 4 h at 70 °C. The embryos were then washed with HM without heparin and tRNA, SSC, and PBST washing series, then incubated with 2 mg/mL bovine serum albumin (BSA) in PBST for 2 h followed by incubation with Anti-DIG-AP Fab fragments (1:5000; Roche, Basel, Switzerland) at 4 °C overnight. After PBST washing, the samples were equilibrated in Alkaline Tris buffer (100 mM Tris-HCl, pH 9.5, 100 mM NaCl, 50 mM MgCl_2_, 0.1% Tween 20) and then incubated in alkaline buffer plus 225 μg/mL nitro-blue tetrazolium chloride (NBT) and 175 μg/mL 5-bromo-4-chloro-3′-indolyphosphate p-toluidine salt (BCIP) for signal detection.

### 4.5. Whole Mount Immunofluorescence

To perform whole-mount immunofluorescence with acetylated α-tubulin (T7451, Sigma, St. Louis, MO, USA), 4 dpf larvae were fixed in Dent’s solution (80% methanol in DMSO) overnight at 4 °C. After rehydration with decreasing methanol series (75%, 50%, and 25%) in PBS, the samples were bleached with bleach solution (0.05 g KOH in 9 mL PBST and 1 mL H_2_O_2_), permeabilized with proteinase K (10 μg/mL), post-fixed with 4% PFA, and washed twice with IF buffer (1% BSA in PBST). After incubation with blocking solution (10% FBS in IF buffer) for 1 h at room temperature, the samples were incubated with the acetylated α-tubulin in blocking solution overnight at 4 °C. Finally, the larvae were incubated with the Alexa Fluor 488 s antibodies (1:500, A10680, Invitrogen, Carlsbad, CA, USA) in blocking solution for 2 h at room temperature and washed with IF buffer for twice. Measurements of the distance between optic tecta were performed as previously described [36,37].

### 4.6. Larval Locomotion Activity Test 

A larval locomotion test was performed as previously described [38]. Free swimming 5 dpf larvae were habituated in 96-well square plates, with one animal per well, in the observation chamber of the Danio Vision tracking system (Noldus, Wageningen, The Netherlands), and videos were recorded for 60 min. After 30 min of habituation in light conditions, each larva was recorded for a total of 30 min with three dark/light cycles, each of which consists of 5 min of dark and 5 min of light. The light intensity was 100 lx and the frame rate was 25/s. The locomotion of each larva was tracked and analyzed by EthoVision XT7 software (Noldus, Wageningen, The Netherlands). For analysis of the locomotor activity, raw data were converted into the velocity (cm/s) of each larva per 30 s time-bin. 

### 4.7. Sleep and Waking Activity of Zebrafish Larvae

The sleep and waking activity were measured as previously described [39,40,41]. Free-swimming 4.5 dpf larvae were habituated in 96-well square plates, with one larva per well, in the observation chamber of the Danio Vision tracking system for one night. After 10 h of habituation, each larva was recorded under controlled lighting conditions (10 h of dark and 14 h of light cycles) from 5 dpf. Six or more consecutive seconds of non-movement were classified as sleep, and all other bouts were classified as being awake. Sleep bouts, sleep bout durations, the total sleep time, average sleep (sleep ratio), sleep latency, and average waking activity were calculated. 

### 4.8. Open Field Test

All behavioral experiments of adult zebrafish were conducted during the same time frame each day. The adult zebrafish used for behavioral tests were 6-month-old male fish. The open field test was performed as previously described [40]. The open field test was performed in a water tank of 20 × 20 × 10 cm. Individual fish were placed in the center zone and allowed to freely swim inside the tank. Videos were recorded from the top viewpoint of the tank for 15 min. The distance moved, the average velocity, and the time spent in the center zone and the peripheral zone were calculated. 

To analyze repetitive and stereotyped behaviors, we conducted the open field test in a zebrafish home tank with a 10 L volume. Videos were recorded for 12 min after 5 min of habituation. In this test, we used a double-blind method to evaluate the swimming patterns within each 15 s. Repetitive behaviors including walling, cornering, small circling, and big circling were scored.

### 4.9. Novel Tank Assay

The novel tank test was performed according to a previously described protocol [42]. Each zebrafish was placed in a transparent tank of 28 × 20 × 5 cm dimensions. The backside of the tank was covered with a light plate to aid video recording. The tester fish was dropped into the tank from the top. Videos were recorded for 12 min from the lateral viewpoint of the tank. For data analysis, we artificially divided the tank into top, middle, and bottom zones. The time spent in each zone as well as the freezing time during the test were quantified. 

### 4.10. Light Dark Box Assay

The light–dark preference test was performed based on a protocol modified from a previous study [43]. The light-and-dark box test was performed in a water tank with inner dimensions measuring 21 × 10 × 7.5 cm. In this experiment, half of the mating tank was taped with black tape and the other half was illuminated. Video recordings started after an acclimation time of 5 min, for the tester fish to explore the tank. Videos were recorded from the top viewpoint of the tank for 6 min. The frequency entering into the light zone and the total freezing time in the light zone were quantified.

### 4.11. Social Interaction Assay

The social interaction assay was performed in a transparent tank divided into 3 sections with two transparent barriers as previously described [44]. A group of three zebrafish was placed in the left side, and a single tester zebrafish was placed in the middle section. The right side of the tank was kept empty. The second section with dimensions measuring 20 ×15 × 13 cm was further divided into three equal-sized subzones; the zone nearest to the social cue was designated “social zone”, the second zone was designated “middle zone”, and the third zone was designated “empty zone”. Behavioral recordings typically started after an acclimation of 5 min. Videos were recorded from the lateral viewpoint of the tank for 15 min. The time spent in each zone was then calculated.

### 4.12. Mirror Assay

The mirror test was performed as the protocol was modified from a previous study [45]. The tank was transparent and with dimensions measuring 28 × 20 × 5 cm. The backside of the tank was covered with a light plate to aid data recording. A mirror was inserted into one side of the tank, and the region in which the tester fish could touch the mirror was designated as a “mirror zone” (3 cm in width, the same to the average body length of adult zebrafish). Videos were recorded from the lateral viewpoint of the tank for 15 min. The time spent in the “mirror zone” was calculated.

### 4.13. Shoaling Assay

The zebrafish is a schooling fish that typically prefers to stay close to its congeners [45,46]. For the shoaling assay, 8 six-month-old zebrafish were placed together in a transparent tank with dimensions measuring 40 × 40 × 15 cm and monitored by video tracking as previously described [33]. Video was recorded for 5 min after a 25 min adaptive phase. The distance between any two zebrafish was measured and analyzed. 

### 4.14. Statistical Analysis

Statistical analysis was performed using GraphPad Prism 7 software (GraphPad, La Jolla, CA, USA). In all experiments, comparisons between *katnal2^+/+^* and *katnal2^-/-^* zebrafish were performed with two-tailed unpaired Student’s *t*-tests. Data are presented as mean ± S.E.M. In all tests, *p* < 0.05 was considered to be statistically significant. In the figures, * indicates *p* < 0.05, ** indicates *p* < 0.01, *** indicates *p* < 0.001, and ns indicates no significance.

## 5. Conclusions

In this study, we generated and characterized a *katnal2* knockout allele in zebrafish. The *katnal2^-/-^* zebrafish displayed autism-like behavioral characteristics as well as developmental delay. These results established a causative relationship between *katnal2* loss and behavior defects, providing supporting evidence for the association between the KATNAL2 variants and ASD in humans.

## Data Availability

Not applicable.

## References

[1] Iakoucheva L.M., Muotri A.R., Sebat J. (2019). Getting to the Cores of Autism. Cell.

[2] Lord C., Brugha T.S., Charman T., Cusack J., Dumas G., Frazier T., Jones E.J.H., Jones R.M., Pickles A., State M.W. (2020). Autism spectrum disorder. Nat. Rev. Dis. Primers.

[3] Baxter A.J., Brugha T.S., Erskine H.E., Scheurer R.W., Vos T., Scott J.G. (2015). The epidemiology and global burden of autism spectrum disorders. Psychol. Med..

[4] Bai D., Yip B.H.K., Windham G.C., Sourander A., Francis R., Yoffe R., Glasson E., Mahjani B., Suominen A., Leonard H. (2019). Association of Genetic and Environmental Factors With Autism in a 5-Country Cohort. JAMA Psychiatry.

[5] Vorstman J.A.S., Parr J.R., Moreno-De-Luca D., Anney R.J.L., Nurnberger Jr J.I., Hallmayer J.F. (2017). Autism genetics: Opportunities and challenges for clinical translation. Nature Rev. Genet..

[6] O’Roak B.J., Vives L., Girirajan S., Karakoc E., Krumm N., Coe B.P., Levy R., Ko A., Lee C., Smith J.D. (2012). Sporadic autism exomes reveal a highly interconnected protein network of de novo mutations. Nature.

[7] Sanders S.J., Murtha M.T., Gupta A.R., Murdoch J.D., Raubeson M.J., Willsey A.J., Ercan-Sencicek A.G., DiLullo N.M., Parikshak N.N., Stein J.L. (2012). De novo mutations revealed by whole-exome sequencing are strongly associated with autism. Nature.

[8] De Rubeis S., He X., Goldberg A.P., Poultney C.S., Samocha K., Ercument Cicek A., Kou Y., Liu L., Fromer M., Walker S. (2014). Synaptic, transcriptional and chromatin genes disrupted in autism. Nature.

[9] Stessman H.A.F., Xiong B., Coe B.P., Wang T., Hoekzema K., Fenckova M., Kvarnung M., Gerdts J., Trinh S., Cosemans N. (2017). Targeted sequencing identifies 91 neurodevelopmental-disorder risk genes with autism and developmental-disability biases. Nat. Genet..

[10] Aspromonte M.C., Bellini M., Gasparini A., Carraro M., Bettella E., Polli R., Cesca F., Bigoni S., Boni S., Carlet O. (2019). Characterization of intellectual disability and autism comorbidity through gene panel sequencing. Hum. Mutat..

[11] Guo H., Wang T., Wu H., Long M., Coe B.P., Li H., Xun G., Ou J., Chen B., Duan G. (2018). Inherited and multiple de novo mutations in autism/developmental delay risk genes suggest a multifactorial model. Mol. Autism.

[12] Husson T., Lecoquierre F., Cassinari K., Charbonnier C., Quenez O., Goldenberg A., Guerrot A.-M., Richard A.-C., Drouin-Garraud V., Brehin A.-C. (2020). Rare genetic susceptibility variants assessment in autism spectrum disorder: Detection rate and practical use. Transl. Psychiatry.

[13] Iossifov I., O’Roak B.J., Sanders S.J., Ronemus M., Krumm N., Levy D., Stessman H.A., Witherspoon K.T., Vives L., Patterson K.E. (2014). The contribution of de novo coding mutations to autism spectrum disorder. Nature.

[14] Wang T., Hoekzema K., Vecchio D., Wu H., Sulovari A., Coe B.P., Gillentine M.A., Wilfert A.B., Perez-Jurado L.A., Kvarnung M. (2020). Large-scale targeted sequencing identifies risk genes for neurodevelopmental disorders. Nat. Commun..

[15] Yuen R.K.C., Thiruvahindrapuram B., Merico D., Walker S., Tammimies K., Hoang N., Chrysler C., Nalpathamkalam T., Pellecchia G., Liu Y. (2015). Whole-genome sequencing of quartet families with autism spectrum disorder. Nat. Med..

[16] Ververis A., Christodoulou A., Christoforou M., Kamilari C., Lederer C.W., Santama N. (2016). A novel family of katanin-like 2 protein isoforms (KATNAL2), interacting with nucleotide-binding proteins Nubp1 and Nubp2, are key regulators of different MT-based processes in mammalian cells. Cell. Mol. Life Sci..

[17] Willsey H.R., Walentek P., Exner C.R.T., Xu Y., Lane A.B., Harland R.M., Heald R., Santama N. (2018). Katanin-like protein Katnal2 is required for ciliogenesis and brain development in Xenopus embryos. Dev. Biol..

[18] Joachimiak E., Waclawek E., Niziolek M., Osinka A., Fabczak H., Gaertig J., Wloga D. (2020). The LisH Domain-Containing N-Terminal Fragment is Important for the Localization, Dimerization, and Stability of Katnal2 in Tetrahymena. Cells.

[19] Mariella A. M. (2013). Franker, Casper C. Hoogenraad. Microtubule-based transport—Basic mechanisms, traffic rules and role in neurological pathogenesis. J. Cell Sci..

[20] Williams M.R., Fricano-Kugler C.J., Getz S.A., Skelton P.D., Lee J., Rizzuto C.P., Geller J.S., Li M., Luikart B.W. (2016). A Retroviral CRISPR-Cas9 System for Cellular Autism-Associated Phenotype Discovery in Developing Neurons. Sci. Rep..

[21] Banks G., Lassi G., Hoerder-Suabedissen A., Tinarelli F., Simon M.M., Wilcox A., Lau P., Lawson T.N., Johnson S., Rutman A. (2018). A missense mutation in Katnal1 underlies behavioural, neurological and ciliary anomalies. Mol. Psychiatry.

[22] Bartholdi D., Stray-Pedersen A., Azzarello-Burri S., Kibaek M., Kirchhoff M., Oneda B., Rødningen O., Schmitt-Mechelke T., Rauch A., Kjaergaard S. (2014). A newly recognized 13q12.3 microdeletion syndrome characterized by intellectual disability, microcephaly, and eczema/atopic dermatitis encompassing the HMGB1 and KATNAL1 genes. Am. J. Med. Genet. Part A.

[23] Mishra-Gorur K., Çağlayan A.O., Schaffer A.E., Chabu C., Henegariu O., Vonhoff F., Akgümüş G.T., Nishimura S., Han W., Tu S. (2014). Mutations in KATNB1 Cause Complex Cerebral Malformations by Disrupting Asymmetrically Dividing Neural Progenitors. Neuron.

[24] Cheung K., Senese S., Kuang J., Bui N., Ongpipattanakul C., Gholkar A., Cohn W., Capri J., Whitelegge J.P., Torres J.Z. (2016). Proteomic Analysis of the Mammalian Katanin Family of Microtubule-severing Enzymes Defines Katanin p80 subunit B-like 1 (KATNBL1) as a Regulator of Mammalian Katanin Microtubule-severing. Mol. Cell. Proteom. MCP.

[25] Solnica-Krezel L., Driever W. (1994). Microtubule arrays of the zebrafish yolk cell: Organization and function during epiboly. Development.

[26] Koh A., Tao S., Jing Goh Y., Chaganty V., See K., Purushothaman K., Orbán L., Mathuru A.S., Wohland T., Winkler C. (2020). A Neurexin2aa deficiency results in axon pathfinding defects and increased anxiety in zebrafish. Hum. Mol. Genet..

[27] Kalueff A.V., Stewart A.M., Gerlai R. (2014). Zebrafish as an emerging model for studying complex brain disorders. Trends Pharmacol. Sci..

[28] Dasgupta S., Simonich M.T., Tanguay R.L., Zhu H., Xia M. (2022). Zebrafish Behavioral Assays in Toxicology. High-Throughput Screening Assays in Toxicology.

[29] Orger M.B., de Polavieja G.G. (2017). Zebrafish Behavior: Opportunities and Challenges. Annu. Rev. Neurosci..

[30] Peng W., Wu Z., Song K., Zhang S., Li Y., Xu M. (2020). Regulation of sleep homeostasis mediator adenosine by basal forebrain glutamatergic neurons. Science.

[31] Hoffman E.J., Turner K.J., Fernandez J.M., Cifuentes D., Ghosh M., Ijaz S., Jain R.A., Kubo F., Bill B.R., Baier H. (2016). Estrogens Suppress a Behavioral Phenotype in Zebrafish Mutants of the Autism Risk Gene, CNTNAP2. Neuron.

[32] Kokel D., Rennekamp A.J., Shah A.H., Liebel U., Peterson R.T. (2012). Behavioral barcoding in the cloud: Embracing data-intensive digital phenotyping in neuropharmacology. Trends Biotechnol..

[33] Hwang W.Y., Fu Y., Reyon D., Maeder M.L., Tsai S.Q., Sander J.D., Peterson R.T., Yeh J.R.J., Joung J.K. (2013). Efficient genome editing in zebrafish using a CRISPR-Cas system. Nat. Biotechnol..

[34] Mali P., Yang L., Esvelt Kevin M., Aach J., Guell M., DiCarlo James E., Norville Julie E., Church George M. (2013). RNA-Guided Human Genome Engineering via Cas9. Science.

[35] Thisse C., Thisse B. (2008). High-resolution in situ hybridization to whole-mount zebrafish embryos. Nat. Protoc..

[36] Wilson S.W., Ross L.S., Parrett T., Easter S.S. (1990). The development of a simple scaffold of axon tracts in the brain of the embryonic zebrafish, Brachydanio rerio. Development.

[37] Bernier R., Golzio C., Xiong B., Stessman H.A., Coe B.P., Penn O., Witherspoon K., Gerdts J., Baker C., Vulto-van Silfhout A.T. (2014). Disruptive CHD8 Mutations Define a Subtype of Autism Early in Development. Cell.

[38] Liu C.-x., Li C.-y., Hu C.-c., Wang Y., Lin J., Jiang Y.-h., Li Q., Xu X. (2018). CRISPR/Cas9-induced shank3b mutant zebrafish display autism-like behaviors. Mol. Autism.

[39] Rihel J., Prober D.A., Schier A.F., Detrich H.W., Westerfield M., Zon L.I. (2010). Chapter 11—Monitoring Sleep and Arousal in Zebrafish. Methods in Cell Biology.

[40] Lee D.A., Oikonomou G., Prober D.A. (2022). Large-scale Analysis of Sleep in Zebrafish. Bio-Protocol.

[41] Rihel J., Prober D.A., Arvanites A., Lam K., Zimmerman S., Jang S., Haggarty S.J., Kokel D., Rubin L.L., Peterson R.T. (2010). Zebrafish Behavioral Profiling Links Drugs to Biological Targets and Rest/Wake Regulation. Science.

[42] Cachat J., Stewart A., Grossman L., Gaikwad S., Kadri F., Chung K.M., Wu N., Wong K., Roy S., Suciu C. (2010). Measuring behavioral and endocrine responses to novelty stress in adult zebrafish. Nat. Protoc..

[43] Maximino C., de Brito T.M., Colmanetti R., Pontes A.A.A., de Castro H.M., de Lacerda R.I.T., Morato S., Gouveia A. (2010). Parametric analyses of anxiety in zebrafish scototaxis. Behavioural Brain Res..

[44] Engeszer R.E., Ryan M.J., Parichy D.M. (2004). Learned Social Preference in Zebrafish. Curr. Biol..

[45] Gerlai R., Lahav M., Guo S., Rosenthal A. (2000). Drinks like a fish: Zebra fish (Danio rerio) as a behavior genetic model to study alcohol effects. Pharmacol. Biochem. Behav..

[46] Green J., Collins C., Kyzar E.J., Pham M., Roth A., Gaikwad S., Cachat J., Stewart A.M., Landsman S., Grieco F. (2012). Automated high-throughput neurophenotyping of zebrafish social behavior. J. Neurosci. Methods.

